# Transactions of the Homœopathic Medical Society of New York

**Published:** 1892-06

**Authors:** 


Transactions of The Homœopathic Medical Society of the State of New York for the year 1891. Volume XXVI. Edited by the Secretary, John L. Moffat, M. D.

These transactions give an account of the fortieth annual meeting of the New York State Society, and will surely be welcomed by every member, giving, as they do, the full proceedings of the meeting at Albany, February 10th and 11th, 1891. It is a book of some 450 pages, and does great credit to the Secretary, Dr. John L. Moffat.

On opening the book we find a portrait of the President for 1892, Dr. F. Park Lewis. President Dr. Geo. W. Dillow's address is full of thought, and gives a general outline of the work before the Convention. The different bureaus are well represented, and many of the papers are quite interesting.

There is one paper by Dr. W. M. Decker to which we wish to take exception. Indeed, it would make good reading matter for an allopathic journal, but it is entirely out of place in the proceedings of an Homœopathic Society, The title is, "It is hazardous to prescribe without a diagnosis." After lauding Pathology to the skies, and claiming Bacteriology to be an exact science, he asks: "What becomes of all the theories of the past?" "No freaks of imagination, or cunning interpretation of Hahnemann's psoric theory of disease can make it harmonize with microbic diseases." Doctor, what homœopathic physician ever attempted to harmonize Hahnemann's psoric theory of disease with the modern microbe theory?

"When Hahnemann did not know what was the matter with his patients, or could not cure them, he diagnosed psora, developed the itch, and then cured it. In this particular he was like that doctor who threw his patient into fits, for he could cure fits." Poor old Hahnemann, had you only lived in this enlightened nineteenth century, you would have been declared a fit subject for the lunatic aslyum by one of your disciples, a so-called homœopathic physician. God save the mark!

The Doctor rages on thus: "Can it be possible that our school of medicine will longer persist in harboring such untruth, such nonsense, in the bright light, the purer light, the microscopical light which characterizes the close of the nineteenth century?" Yes, Doctor, in spite of all the pathological, bacteriological, and microscopical light of the times there are, bless God! physicians all over the world that believe in Hahnemann simply because they have found his theories true, and daily exemplify them in the cure of disease.

"We need more Sulphur and Brimstone, too, in order to exterminate the itch in the Homœopathic School of Medicine. Our school is the only medical school in the world that is infested with psora. We need to be disinfected. It is high time to clean house, and get rid of that which is offensive to others and detrimental to ourselves" Just so. The best remedies to clean house with are cathartics. Give the patient a good physic. Kill the microbes, and the patient will get well. Should the patient perchance die, well let it be. The good Doctor, in killing the patient, surely killed the microbes also. This killing the bacilli, microbes, *et id omne genus* is the main point. What crude, materialistic ideas some homœopathic physicians have of disease! W. S

